# Supplementary figures and images for: Gene regulation by a protein translation factor at the single-cell level
Source: PLoS Comput Biol. 2022 May 6;18(5):e1010087. doi: 10.1371/journal.pcbi.1010087 (PMC9116677; doi:10.1371/journal.pcbi.1010087)

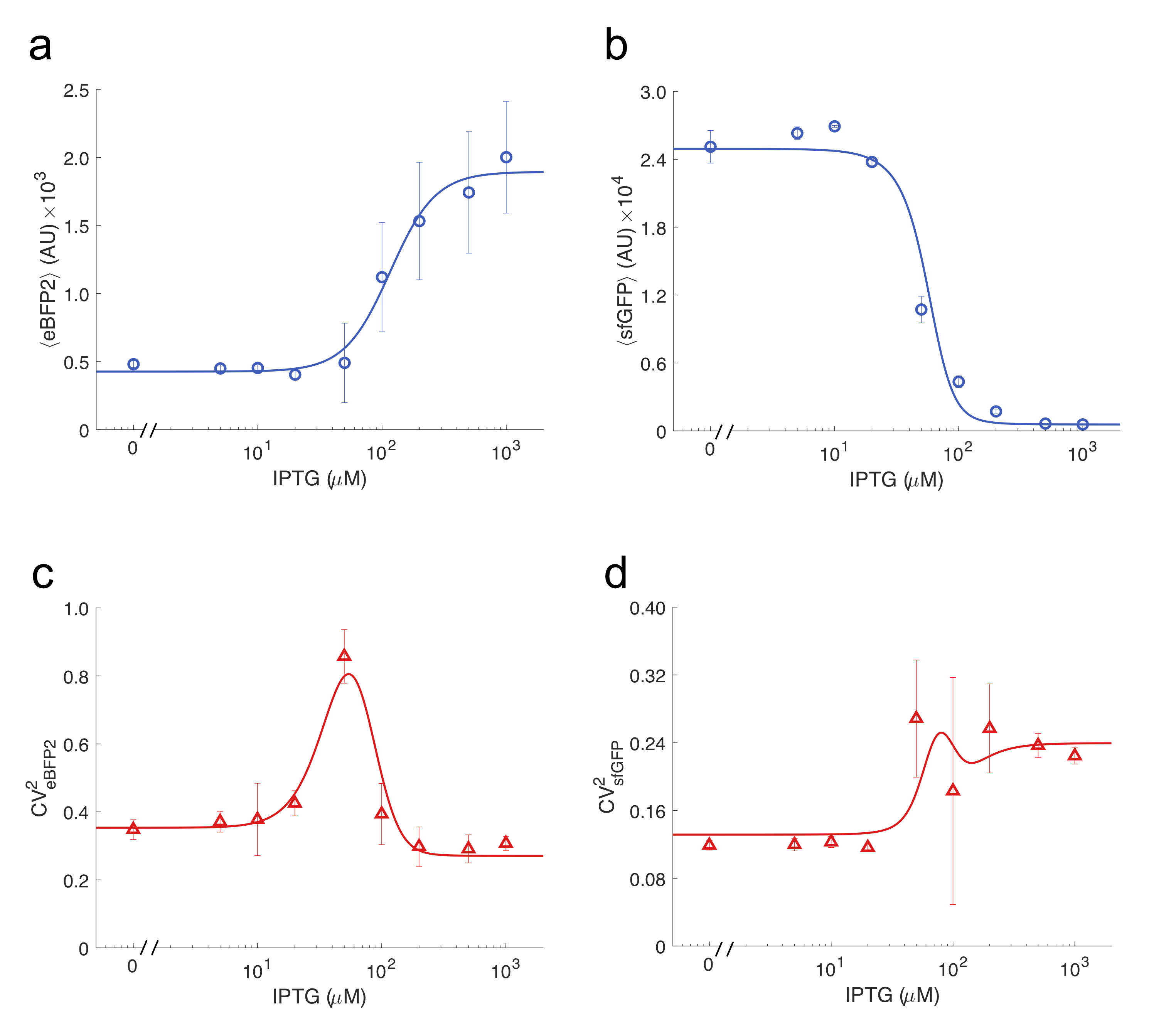

Supplement: S1 Fig — a) Mean of eBFP2 expression as a function of IPTG. b) Mean of sfGFP expression as a function of IPTG. c) Noise of eBFP2 expression as a function of IPTG. d) Noise of sfGFP expression as a function of IPTG. Points correspond to the values of the population shown in the main figures. Error bars correspond to standard errors calculated from four different populations. Solid lines correspond to predictions with the mathematical model. (TIF) [file pcbi.1010087.s001.tif]

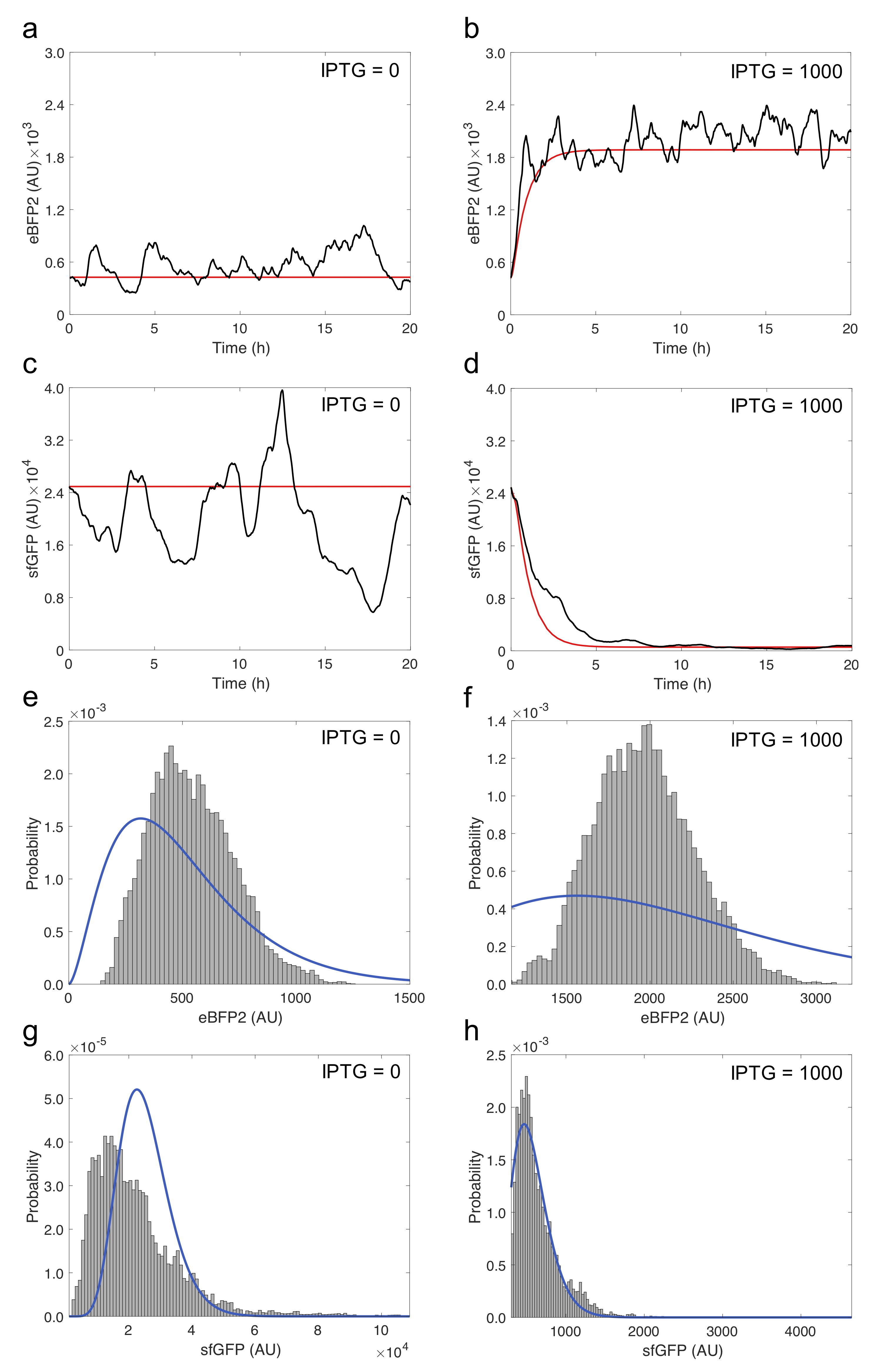

Supplement: S2 Fig — a-d) Stochastic trajectories with time of eBFP2 and sfGFP for two different IPTG concentrations. In red, deterministic trajectories. The initial condition corresponds to the uninduced state in all cases. e-h) Histograms of protein expression computed from long trajectories. The Gamma distributions fitted against the experimental data (blue lines) were also represented. (TIF) [file pcbi.1010087.s002.tif]

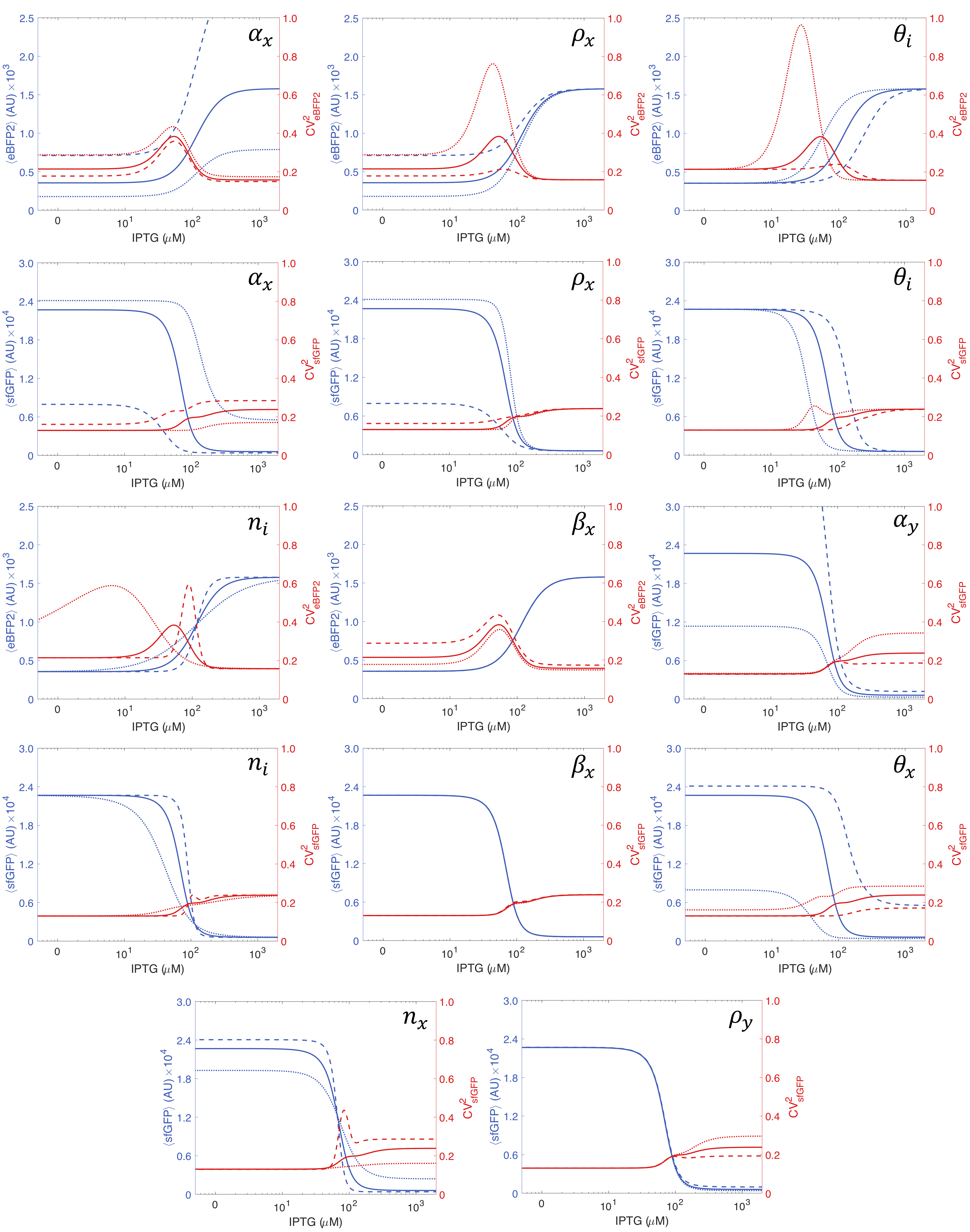

Supplement: S3 Fig — Plots of mean and noise of expression as a function of IPTG, where solid lines correspond to the dynamics predicted with the adjusted parameter, dotted lines to the dynamics if the parameter increases 2-fold, and dashed lines to the dynamics if the parameter decreases 2-fold. (TIF) [file pcbi.1010087.s003.tif]

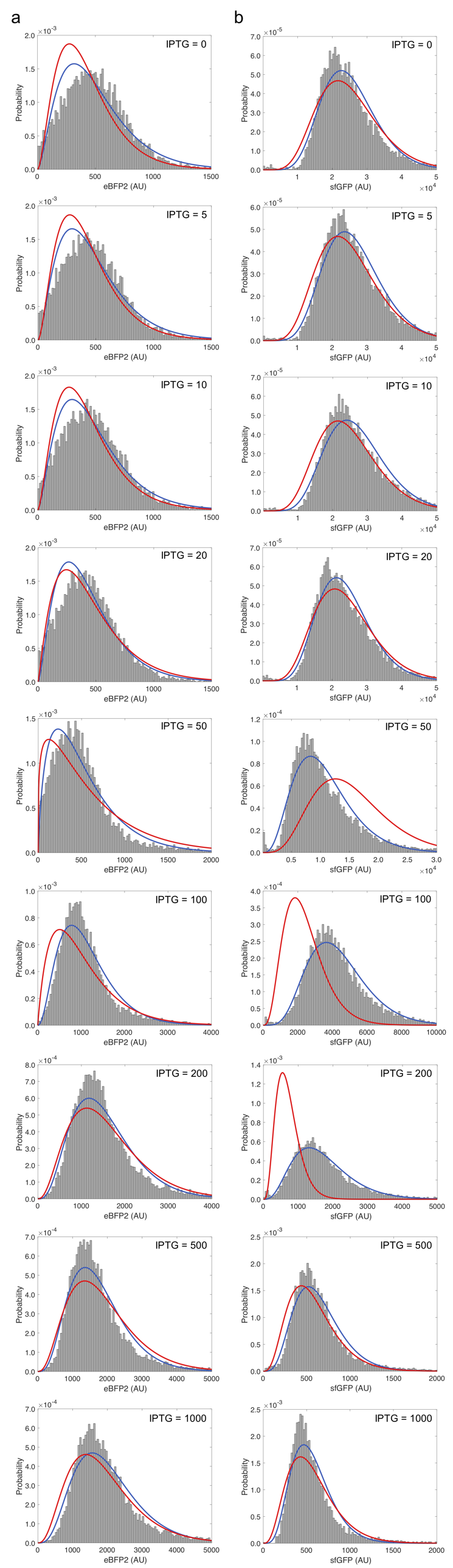

Supplement: S4 Fig — Histograms of experimental single-cell fluorescence for both a) eBFP2 and b) sfGFP for different induction conditions with IPTG, together with fitted Gamma distributions against the data (blue lines) and predicted Gamma distributions obtained by using the model values of mean and noise (red lines). (TIF) [file pcbi.1010087.s004.tif]

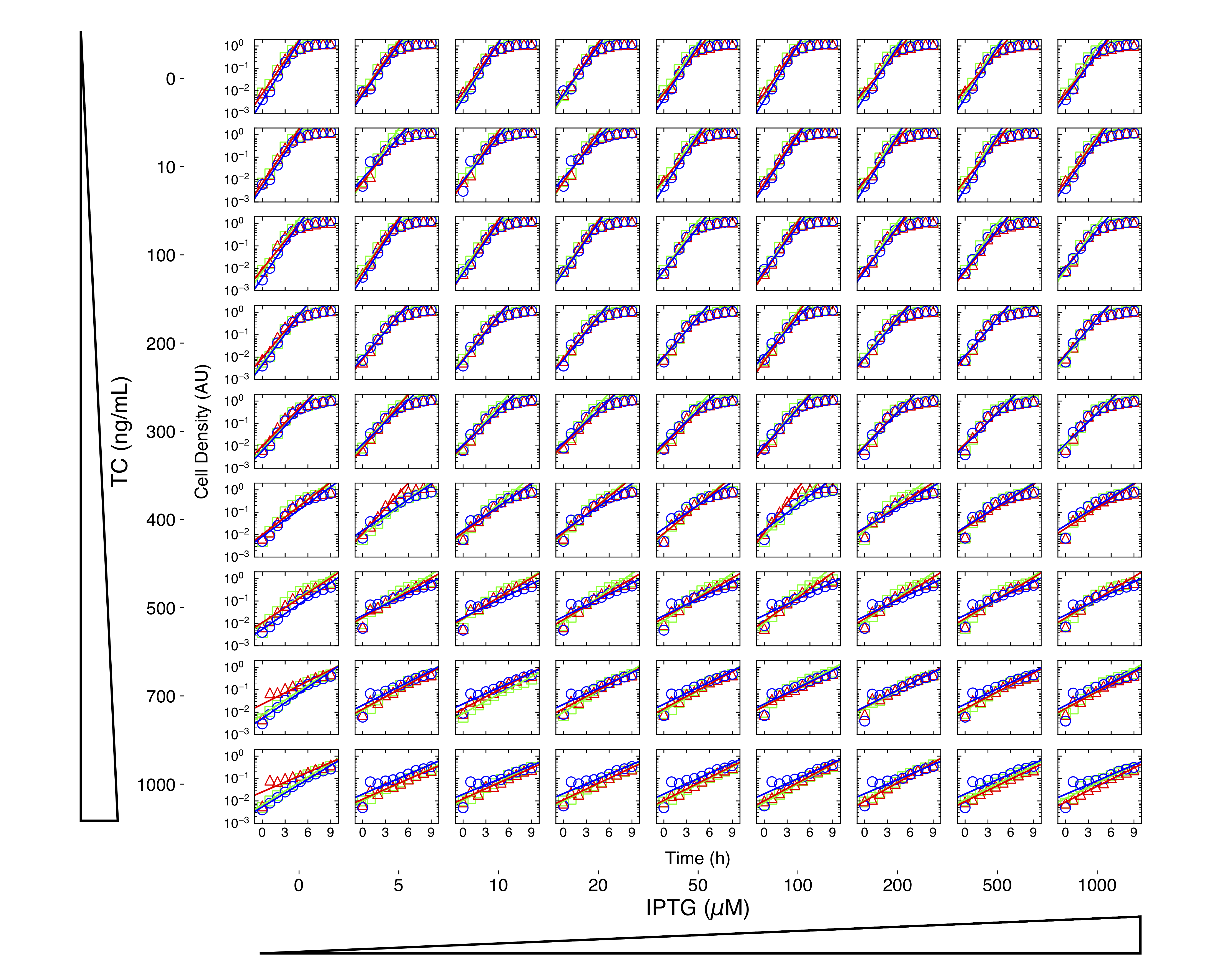

Supplement: S5 Fig — Three different populations (blue, red, and green) were monitored with time. Points correspond to absorbance values, while solid lines come from fitted exponential trends. (TIF) [file pcbi.1010087.s005.tif]

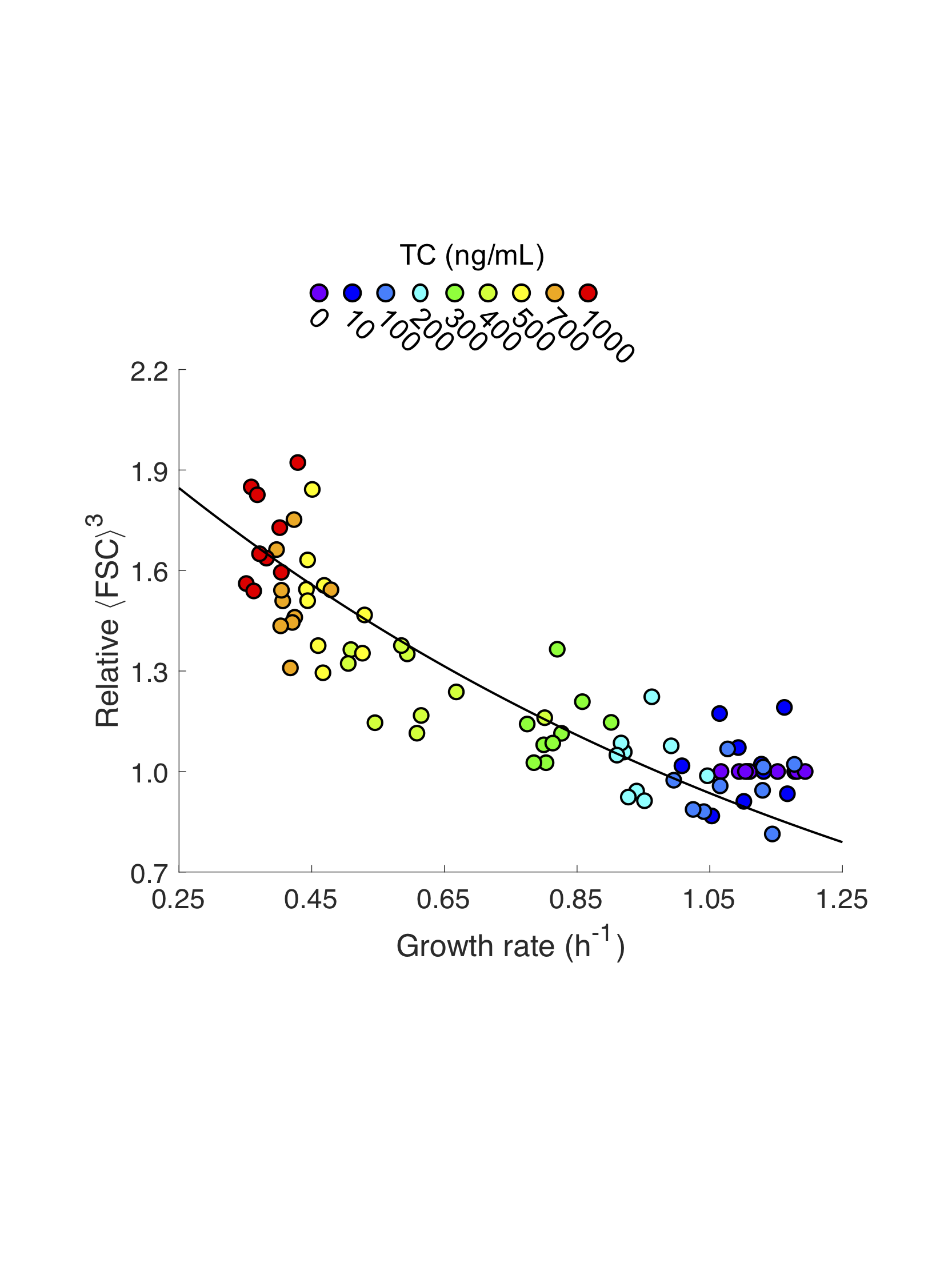

Supplement: S6 Fig — a) Schematics to show that as TC increases, cells grow slower and are bigger. b) Scatter plot between the cube of the forward scattering signal (proxy of cellular volume) and the growth rate for the 81 IPTG and TC conditions (colored by TC condition). An exponential trend was adjusted (solid line). (TIF) [file pcbi.1010087.s006.tif]
