# Supplementary material for: Gene regulation by a protein translation factor at the single-cell level
Source: PLoS Comput Biol. 2022 May 6;18(5):e1010087. doi: 10.1371/journal.pcbi.1010087 (PMC9116677; doi:10.1371/journal.pcbi.1010087)
Supplement: S1 Appendix — Derivation of the mathematical expressions of noise in eBFP2 and sfGFP having followed a Langevin formalism and the mean-field approximation. (DOCX) [file pcbi.1010087.s007.docx]

The differential equations for eBFP2 expression are

$$\frac{d\left[ \text{mRNA}_{\text{eBFP2}} \right]}{dt}=\psi_{x}\left( \frac{\rho_{x}+\left( \frac{\text{IPTG}}{\theta_{i}} \right)^{n_{i}}}{1+\left( \frac{\text{IPTG}}{\theta_{i}} \right)^{n_{i}}} \right)-\delta\left[ \text{mRNA}_{\text{eBFP2}} \right] ,$$

$$\frac{d\left[ \text{eBFP2} \right]}{dt}=\phi_{x}\left[ \text{mRNA}_{\text{eBFP2}} \right]-\mu\left[ \text{eBFP2} \right] ,$$

where $\psi_{x}$ is the maximal transcription rate, $\phi_{x}$ the translation rate, and $\delta$ the mRNA degradation rate. The rest of the parameters are defined in the main text. Note that $\alpha_{x}=\frac{\psi_{x}\phi_{x}}{\delta\mu}$. The steady state solutions are

$$\left\langle\text{mRNA}_{\text{eBFP2}} \right\rangle=\frac{\psi_{x}}{\delta}\left( \frac{\rho_{x}+\left( \frac{\text{IPTG}}{\theta_{i}} \right)^{n_{i}}}{1+\left( \frac{\text{IPTG}}{\theta_{i}} \right)^{n_{i}}} \right) ,$$

$$\left\langle\text{eBFP2} \right\rangle=\frac{\phi_{x}}{\mu}\left\langle\text{mRNA}_{\text{eBFP2}} \right\rangle=\alpha_{x}\left( \frac{\rho_{x}+\left( \frac{\text{IPTG}}{\theta_{i}} \right)^{n_{i}}}{1+\left( \frac{\text{IPTG}}{\theta_{i}} \right)^{n_{i}}} \right) .$$

Denoting by $\Delta\left[ \text{mRNA}_{\text{eBFP2}} \right]=\left[ \text{mRNA}_{\text{eBFP2}} \right]-\langle\text{mRNA}_{\text{eBFP2}}\rangle$ and $\Delta\left[ \text{eBFP2} \right]=\left[ \text{eBFP2} \right]-\left\langle\text{eBFP2} \right\rangle$ the fluctuations around the steady state, the stochastic differential equations according to the Langevin formalism read

$$\frac{d\Delta\left[ \text{mRNA}_{\text{eBFP2}} \right]}{dt}=-\delta\Delta\left[ \text{mRNA}_{\text{eBFP2}} \right]+q_{x,\text{ex}}\xi_{x,\text{ex}}\left( t \right)+q_{x,\text{in}}\xi_{x,\text{in}}\left( t \right)+\psi_{x}\left( \frac{n_{i}\left( 1-\rho_{x} \right)\cdot\left( \frac{\text{IPTG}}{\theta_{i}} \right)^{n_{i}-1}}{{\theta_{i}\left( 1+\left( \frac{\text{IPTG}}{\theta_{i}} \right)^{n_{i}} \right)}^{2}} \right)\cdot\xi_{\text{lac}}\left( t \right) ,$$

$$\frac{d\Delta\left[ \text{eBFP2} \right]}{dt}=\phi_{x}\Delta\left[ \text{mRNA}_{\text{eBFP2}} \right]-\mu\Delta\left[ \text{eBFP2} \right]$$

where $\xi_{x,\text{ex}}$ denotes the extrinsic noise, $\xi_{x,\text{in}}$ the intrinsic noise, and $\xi_{\text{lac}}$ the noise associated with LacI expression. The noise generated at the level of protein production is neglected, unless the propagated. The statistics of these noises are $\left\langle\xi_{x,\text{in}}(t) \right\rangle=0, \left\langle\xi_{x,\text{in}}\left( 0 \right)\cdot\xi_{x,\text{in}}\left( t \right) \right\rangle=\delta\left( t \right)$, $\left\langle\xi_{x,\text{ex}}(t) \right\rangle=0, \left\langle\xi_{x,\text{ex}}\left( 0 \right)\cdot\xi_{x,\text{ex}}\left( t \right) \right\rangle=\frac{\mu}{2}e^{- \mu\left| t \right|}$, and $\left\langle\xi_{\text{lac}}\left( t \right) \right\rangle=0$, $\left\langle\xi_{\text{lac}}\left( 0 \right)\cdot\xi_{\text{lac}}\left( t \right) \right\rangle=\eta_{\text{lac}}^{2}e^{- \mu\left| t \right|}$. Also, $q_{x,\text{in}}$, $q_{x,\text{ex}}$, and $\eta_{\text{lac}}$ denote noise amplitudes. The extrinsic noise amplitude can be written as

$$q_{x,\text{ex}}\text{=}\frac{C_{x,\text{ex}}}{\sqrt{\mu}}\left\langle\text{mRNA}_{\text{eBFP2}} \right\rangle$$

and the intrinsic noise amplitude as

$$q_{x,\text{in}}\text{= }\sqrt{\psi_{x}\left( \frac{\rho_{x}+\left( \frac{\text{IPTG}}{\theta_{i}} \right)^{n_{i}}}{1+\left( \frac{\text{IPTG}}{\theta_{i}} \right)^{n_{i}}} \right)+\delta\langle\text{mRNA}_{\text{eBFP2}}\rangle}=\sqrt{2\delta\langle\text{mRNA}_{\text{eBFP2}}\rangle} .$$

Applying the Fourier transform (assuming independence among the different stochastic processes, knowing that this is not necessarily true due to the coupling by the extrinsic noise), the spectral powers of the fluctuations read

$$S_{\Delta\left[ \text{mRNA}_{\text{eBFP2}} \right]}=\frac{C_{x,\text{ex}}^{2}{\mu\langle\text{mRNA}_{\text{eBFP2}}\rangle}^{2}}{\left( \omega^{2}+\delta^{2} \right)\cdot(\omega^{2}+\mu^{2})}+\frac{2\delta_{x}\left\langle\text{mRNA}_{\text{eBFP2}} \right\rangle}{\omega^{2}+\delta^{2}}+\frac{\psi_{x}^{2}}{\omega^{2}+\delta^{2}}\left( \frac{n_{i}\left( 1-\rho_{x} \right)\cdot\left( \frac{\text{IPTG}}{\theta_{i}} \right)^{n_{i}-1}}{{\theta_{i}\left( 1+\left( \frac{\text{IPTG}}{\theta_{i}} \right)^{n_{i}} \right)}^{2}} \right)^{2}\cdot\frac{2\mu\eta_{\text{lac}}^{2}}{\omega^{2}+\mu^{2}} ,$$

$$S_{\text{Δ}[\text{eBFP2] }}=\frac{\phi_{x}^{2}}{\omega^{2}+\mu^{2}}S_{\Delta\left[ \text{mRNA}_{\text{eBFP2}} \right]} .$$

Applying the Wiener-Khinchin theorem (in the limit $\mu\ll\delta$), we obtain the expression for the total noise of eBFP2

$$\text{CV}_{\text{eBFP2}}^{2}=\frac{\left\langle\Delta\left[ \text{eBFP2} \right]^{2} \right\rangle}{\left\langle\text{eBFP2} \right\rangle^{2}}=\eta_{x}^{2}+\frac{\beta_{x}}{\langle\text{eBFP2}\rangle}+{\frac{1}{2}\left( \frac{\alpha_{x}n_{i}\left( 1-\rho_{x} \right)\cdot\left( \frac{\text{IPTG}}{\theta_{i}} \right)^{n_{i}-1}}{\theta_{i}\left( 1+\left( \frac{\text{IPTG}}{\theta_{i}} \right)^{n_{i}} \right)^{2}} \right)}^{2}\frac{\eta_{\text{lac}}^{2}}{\left\langle\text{eBFP2} \right\rangle^{2}} ,$$

where $\eta_{x}^{2}=\frac{C_{x,\text{ex}}^{2}}{4\delta^{2}}$ and $\beta_{x}=\frac{\phi_{x}}{\delta}$.

In addition, the differential equations for sfGFP expression are

$$\frac{d\left[ \text{mRNA}_{\text{sfGFP}} \right]}{dt}=\psi_{y}-\delta\left[ \text{mRNA}_{\text{sfGFP}} \right] ,$$

$$\frac{d\left[ \text{sfGFP} \right]}{dt}=\phi_{y}\left( \frac{1+\rho_{y}\left( \frac{\left[ \text{eBFP2} \right]}{\theta_{x}} \right)^{n_{x}}}{1+\left( \frac{\left[ \text{eBFP2} \right]}{\theta_{x}} \right)^{n_{x}}} \right)\left[ \text{mRNA}_{\text{sfGFP}} \right]-\mu\left[ \text{sfGFP} \right] ,$$

where $\psi_{y}$ is the transcription rate and $\phi_{y}$ the maximal translation rate. The rest of the parameters are defined in the main text. Note that $\alpha_{y}=\frac{\psi_{y}\phi_{y}}{\delta\mu}$. The steady state solutions are

$$\left\langle\text{mRNA}_{\text{sfGFP}} \right\rangle=\frac{\psi_{y}}{\delta} ,$$

$$\left\langle\text{sfGFP} \right\rangle=\frac{\phi_{y}}{\mu}\left( \frac{1+\rho_{y}\left( \frac{\left\langle\text{eBFP2} \right\rangle}{\theta_{x}} \right)^{n_{x}}}{1+\left( \frac{\left\langle\text{eBFP2} \right\rangle}{\theta_{x}} \right)^{n_{x}}} \right)\left\langle\text{mRNA}_{\text{sfGFP}} \right\rangle=\alpha_{y}\left( \frac{1+\rho_{y}\left( \frac{\left\langle\text{eBFP2} \right\rangle}{\theta_{x}} \right)^{n_{x}}}{1+\left( \frac{\left\langle\text{eBFP2} \right\rangle}{\theta_{x}} \right)^{n_{x}}} \right) .$$

Since the mRNA is in this case actively transcribed, we can assume that the noise in mRNA expression is low. In addition, because the regulator acts by repressing translation, we can assume that the noise propagated from the mRNA to the protein is negligible. Denoting by $\Delta\left[ \text{sfGFP} \right]=\left[ \text{sfGFP} \right]-\left\langle\text{sfGFP} \right\rangle$ the fluctuation around the steady state, the stochastic differential equation according to the Langevin formalism reads

$$\frac{d\Delta\left[ \text{sfGFP} \right]}{dt}=-\mu\Delta\left[ \text{sfGFP} \right]+\phi_{y}\left( \frac{n_{x}\left( {1-\rho}_{y} \right)\cdot\left( \frac{\left\langle\text{eBFP2} \right\rangle}{\theta_{x}} \right)^{n_{x}-1}}{\theta_{x}\left( 1+\left( \frac{\left\langle\text{eBFP2} \right\rangle}{\theta_{x}} \right)^{n_{x}} \right)^{2}} \right)\cdot\left\langle\text{mRNA}_{\text{sfGFP}} \right\rangle\cdot\Delta\left[ \text{eBFP2} \right]+q_{y,\text{ex}}\xi_{y,\text{ex}}\left( t \right)+q_{y,\text{in}}\xi_{y,\text{in}}\left( t \right) ,$$

where $\xi_{y,\text{ex}}$ denotes the extrinsic noise and $\xi_{y,\text{in}}$ the intrinsic noise. The statistics of these noises are $\left\langle\xi_{y,\text{in}}(t) \right\rangle=0, \left\langle\xi_{y,\text{in}}\left( 0 \right)\cdot\xi_{y,\text{in}}\left( t \right) \right\rangle=\delta\left( t \right)$, and $\left\langle\xi_{y,\text{ex}}(t) \right\rangle=0, \left\langle\xi_{y,\text{ex}}\left( 0 \right)\cdot\xi_{y,\text{ex}}\left( t \right) \right\rangle=\frac{\mu}{2}e^{- \mu\left| t \right|}$. Also, $q_{y,\text{in}}$ and $q_{y,\text{ex}}$ denote noise amplitudes. The extrinsic noise amplitude can be written as

$$q_{y,\text{ex}}\text{=}C_{y,\text{ex}}\sqrt{\mu}\left\langle\text{sfGFP} \right\rangle$$

and the intrinsic noise amplitude as

$$q_{y,\text{in}}\text{= }\sqrt{2\mu\langle\text{sfGFP}\rangle} .$$

Applying the Fourier transform (assuming independence among the different stochastic processes, knowing that this is not necessarily true due to the coupling by the extrinsic noise), the spectral power of the fluctuation reads

$$S_{\Delta\left[ \text{sfGFP} \right]}=\frac{C_{y,\text{ex}}^{2}{\mu\left\langle\text{sfGFP} \right\rangle}^{2}}{\left( \omega^{2}+\mu^{2} \right)^{2}}+\frac{2\mu\left\langle\text{sfGFP} \right\rangle}{\omega^{2}+\mu^{2}}+\phi_{y}^{2}\left( \frac{n_{x}\left( {1-\rho}_{y} \right)\cdot\left( \frac{\left\langle\text{eBFP2} \right\rangle}{\theta_{x}} \right)^{n_{x}-1}}{\theta_{x}\left( 1+\left( \frac{\left\langle\text{eBFP2} \right\rangle}{\theta_{x}} \right)^{n_{x}} \right)^{2}} \right)^{2}\cdot\left\langle\text{mRNA}_{\text{sfGFP}} \right\rangle^{2}\cdot\frac{S_{\Delta\left[ \text{eBFP2} \right]}}{\left( {\omega^{2}+\mu}^{2} \right)} .$$

If we perform the following approximation

$$S_{\Delta\left[ \text{eBFP2} \right]}\approx\frac{2\mu}{\left( {\omega^{2}+\mu}^{2} \right)}\left\langle\text{eBFP2} \right\rangle^{2}\text{CV}_{\text{eBFP2}}^{2}$$

to facilitate the analytical treatment and we apply the Wiener-Khinchin theorem, we obtain the expression for the total noise of sfGFP

$$\text{CV}_{\text{sfGFP}}^{2}=\eta_{y}^{2}+\frac{\beta_{y}}{\left\langle\text{sfGFP} \right\rangle}+{\frac{1}{2}\left( \frac{\alpha_{y}n_{x}\left( {1-\rho}_{y} \right)\cdot\left( \frac{\left\langle\text{eBFP2} \right\rangle}{\theta_{x}} \right)^{n_{x}-1}}{\theta_{x}\left( 1+\left( \frac{\left\langle\text{eBFP2} \right\rangle}{\theta_{x}} \right)^{n_{x}} \right)^{2}} \right)}^{2}{\frac{{\gamma_{y}\left\langle\text{eBFP2} \right\rangle}^{2}}{\left\langle\text{sfGFP} \right\rangle^{2}}\text{CV}}_{\text{eBFP2}}^{2} ,$$

where $\eta_{y}^{2}=\frac{C_{y,\text{ex}}^{2}}{4}$ and $\beta_{y}=1$ and $\gamma_{y}=1$ if the concentrations are given in number of molecules per cell ($\neq1$ in the case of arbitrary units of fluorescence).
