# Supplementary material for: Gene regulation by a protein translation factor at the single-cell level
Source: PLoS Comput Biol. 2022 May 6;18(5):e1010087. doi: 10.1371/journal.pcbi.1010087 (PMC9116677; doi:10.1371/journal.pcbi.1010087)
Supplement: S2 Appendix — Derivation of the Gamma distribution for protein expression from a general stochastic differential equation. (DOCX) [file pcbi.1010087.s008.docx]

Let us consider the following general stochastic differential equation

$$\frac{dx}{dt}=f\left( x \right)+q\left( x \right)\xi\left( t \right) ,$$

where $\xi$ is a Wiener process and $x$ denotes the concentration of a given protein. Then, the Fokker-Planck equation in steady state reads

$$f\left( x \right)P\left( x \right)=\frac{1}{2}\partial_{x}\left( q^{2}\left( x \right)P\left( x \right) \right) ,$$

where $P\left( x \right)$ is the probability distribution for protein expression. The analytical solution is

$$P\left( x \right)=\frac{C}{q^{2}(x)}e^{\int\frac{2f(x)}{q^{2}(x)} dx} ,$$

where $C$ is a normalization constant. If we consider $f\left( x \right)=\phi-\mu x$, where $\phi$ is the translation rate and $\mu$ the cell growth rate, and $q^{2}\left( x \right)=2\sigma x$, with $\sigma$ a proportionality constant, it turns out that

$$P\left( x \right)=\frac{C}{2\sigma x}e^{\frac{\phi}{\sigma}\ln\left( x \right)-\frac{\mu}{\sigma}x}=\frac{C}{2\sigma} x^{\frac{\phi}{\sigma}-1}e^{-\frac{\mu}{\sigma}x} .$$

Knowing that the Gamma probability distribution reads

$$P\left( x \right)=\frac{1}{b^{a}\Gamma(a)}{x^{a-1}e}^{-x/b} ,$$

where $a$ is the shape parameter and $b$ the scale parameter, we can define $a=\frac{\phi}{\sigma}$, $b=\frac{\sigma}{\mu}$ , and $C=\frac{2\sigma}{b^{a}\Gamma(a)}$ to match the two equations.

It is important to note that this derivation is only valid in the case of white noise (i.e., delta-correlated), as it is the case of the intrinsic noise. However, gene expression is affected by other types of noise in the cell, such as colored noise (i.e., exponentially-correlated), as it is the case of the extrinsic noise or the regulation noise. A colored noise can be generated from a white noise. In this work, we used the Gamma probability distribution to model protein expression, but we are aware that this is an assumption. Further theoretical work might be done to study the probability distribution in the case of a combination of white and colored noises.
