# Supplementary material for: Gene regulation by a protein translation factor at the single-cell level
Source: PLoS Comput Biol. 2022 May 6;18(5):e1010087. doi: 10.1371/journal.pcbi.1010087 (PMC9116677; doi:10.1371/journal.pcbi.1010087)
Supplement: Attachment — Submitted filename: cover letter PCOMPBIOL-D-21-00828.pdf [file pcbi.1010087.s010.pdf]

Valencia, 18 October 2021

Dear Editors of PLoS Computational Biology,

I am pleased to submit a revised version of our manuscript entitled “Gene regulation by a protein translation factor at the single-cell level" (PCOMPBIOL-D-21-00828) for your consideration at PLoS Computational Biology.

Below, I provide a detailed point-by-point response to all reviewers’ comments. I hope both the reviewers and the editors find now our work suitable for publication.

I look forward to hearing from you.

Sincerely yours,

Guillermo Rodrigo, Ph.D.
Institute for Integrative Systems Biology (I2SysBio)
Consejo Superior de Investigaciones Científicas (CSIC)
Universitat de València (UV)
9 Cat. Agustín Escardino, 46980 Paterna, Spain
Phone: +34 963 544 786 (ext. 44786)
E-mail: guillermo.rodrigo@csic.es
Web: https://biosysdesign.csic.es

Reviewer #1

The general idea of the paper is interesting but the execution is lacking on multiple levels including data analysis and presentation.

1. The paper is poorly written in general. It is difficult to grasp the intention of the presented data. There are a multitude of figures but the research direction is not quite clear. The Introduction is very generic and does not articulate sufficiently the importance of the research.

Authors' response: This is a subjective statement that of course we do not share. The Introduction is generic because there is no previous work on stochastic protein expression regulated at the level of translation. In any case, in this revision of our work, we have rewritten some parts of the Introduction to highlight the importance of this research and of the Results to provide a more thorough explanation.

2. Noise buffering by translational regulation should be better defined and articulated. What does "buffering" mean and how is it visible in this paper's data?

Authors' response: In this revision of our work, we provide a more thorough explanation. It is well established that when transcription is low and translation high, noise is higher because translation acts as an amplification layer. However, when transcription is high and translation low, noise is lower. In this regard, we can say that noise is buffered. See e.g. Kaern et al., Nat. Rev. Genet., 2005, 6:451-464 for a review. In the case of this paper, in addition, there is a regulation (repression) at the level of translation.

3. The description of the circuit is almost missing. The description and illustration of molecular elements of the design would help. The same is true about almost all experimental methods. The manuscript should be understandable without reading any of the references.

Authors' response: We believe that this comment is not justified. First, it is not true that the description of the circuit was missing. The first paragraph of the Results section provides such an explanation (lines 93-106, page 6, in previous version). In addition, Fig. 1a provides an illustration of the genetic system, in which the DNA, RNA, and protein layers are shown, together with the regulations exerted by the elements. Also, on top of Fig. 1b,c we provide the sequence and structural details of the cis-regulatory regions (PLlac promoter for transcription regulation and MS2CP RNA motif for transcription regulation). Second, it is not true that the experimental methods were not explained. In the Methods section, we detailed how plasmids were constructed (lines 279-293, page 14, in previous version), how cells were grown (lines 295-303, page 14, in previous version), and how flow cytometry data were acquired (lines 307-322, page 15, in previous version). Finally, we may agree with the reviewer that a manuscript should be understandable without reading the references, although some basic knowledge on previous developments in the field is always required. In this revised version, we have tried to elaborate more some ideas presented.

4. The models are phenomenological - which could be great, but here it is unclear what insights we gain. A proper metric for the noise propagation is not defined although it would help with the analysis of the data since the noise propagation is the main topic.

Authors' response: Our models are phenomenological to some extent. As we state in the manuscript, we used Hill equations to model the deterministic (mean) response. This is a

SCIENTIFIC PARK OF U. VAL.
9 CAT. AGUSTÍN ESCARDINO
46980 PATERNA, SPAIN

normal practice in the field. Then, we performed some calculations in the mean-field limit, as done e.g. in Pedraza & van Oudenaarden, Science, 2005, 307:1965-1969; or Rodrigo, Phys. Rev. E, 2019, 100:032415, to derive analytical expressions for the noise (variation in gene expression from cell to cell). See the new supplementary Appendix S1. The model is instrumental to capture the trend of the data and then it supports the underlying regulatory mechanism. The metric for noise propagation is precisely the term that we named "regulation noise". Total noise in gene expression is the sum of extrinsic noise, intrinsic noise, and regulation noise (which is the noise propagated from gene to gene).

5. The noise is treated very loosely. The description suggests that it relates to stochastic concentration variations but no attempt is made to differentiate that from deterministic effects that contribute to the variation. The propagated noise level might be treated as such but it is quite arbitrary to assume so.

Authors' response: In this work, we performed single-cell fluorescence measurements in several induction conditions by flow cytometry. These measurements provide the distribution of expression for a cell population. The variation from cell to cell is essentially due to molecular noise (non-genetic variability), as the population is clonal in lab conditions. From these data, one can calculate the mean and the standard deviation for each condition. Then, noise can be defined as the coefficient of variation (ratio between standard deviation and mean). This is what we did and, as far as we can tell, this a normal practice in the field to investigate noise in gene expression. Thus, we do not understand the question posed by the reviewer.

6. The trends that are presented on the figures are only roughly matched by simulations and sometimes deviate so strongly that it is difficult to see the similarity. The uncertainty of measurements is not presented.

Authors' response: Overall, we see a good agreement between the model and the data. Of course, a larger discrepancy can appear in some cases, but this just reflects the complexity of the biological system, which cannot be fully explained with simple mathematical equations. We suggest the reviewer to examine much of the previous work in the field to realize about this. Moreover, in our plots, we represent on the one hand the mean, and on the other hand the noise. The noise would be the magnitude of the error bar in a classical plot. The mean and the noise (variability, uncertainty, or whatever name) are calculated from ~10^4 single cell measurements.

7. The most interesting aspect is that Gamma distributions match the trends of fluorescence histograms quite nicely. Although, the reasons of this behavior are not quite clear from the text.

Authors' response: In our manuscript we state that "we considered a Gamma distribution, which has been shown to describe quite well the stochasticity of genetic systems [26] and which emerges from ab initio calculations [27]" (lines 150-152, page 8, in previous version). Ref. 26 is for Taniguchi et al., Science, 2010, 329:533-538; and ref. 27 for Friedman et al., Phys. Rev. Lett., 2006, 97:168302. In addition, for this revision we have included a supplementary Appendix (S2) to show how the Gamma distribution emerges from the Fokker-Planck formulation.

Reviewer #2

In this paper Dolcemascolo et al. study noise propagation in a synthetic genetic network where protein expression is regulated at the level of translation. The authors used the MS2-MCP system to inhibit the translation of a super folding GFP (sfGFP) variant and monitored the expression of both the regulator (MCP fused to enhanced blue fluorescent protein 2 or eBFP2) and the target (sfGFP) in E. coli. MCP was expressed under the control of a lac inducible promoter so that the expression level of MCP and hence the translational repression of sfGFP could be controlled by IPTG treatment. The authors empirically determined the steady state distributions of eBFP2 and sfGFP at different IPTG concentrations as well as under tetracycline treatment that modified the growth rate and various kinetic properties of the cells. The empirical data were analyzed by computing noise defined as the CV^2, where the CV is the coefficient of variation.

The empirical contributions of this paper are strong: a new synthetic gene circuit has been established and produces excellent quantitative data at single-cell resolution.

The computational/theoretical contributions however are rather weak. Although the authors call it "stochastic modeling", equations 6 (in Materials and Methods) do not fit the commonly accepted meaning of the term in the field of stochastic gene regulation and can best be described as deterministic curve fitting. A few observations regarding Equations 6:

Authors' response: One thing is deterministic modeling and another is analytical treatment, which is important to not confuse. Our work models and analyzes our own-generated experimental data from a stochastic perspective, considering both transcription and translation regulation, as explained in the Methods section. We (the corresponding author) have vast experience in mathematical modeling in biology and we cannot accept the comments raised by the reviewer. Our stochastic modeling is state-of-the-art and very appropriate to analyze the experimental data. Proof of this is the overall nice agreement between theory and experiments that we achieve.

1. The third term in the expressions for CV^2 in equation 6 performs error propagation where the standard deviation of a dependent variable is linearly approximated as a function of the standard deviations of its independent variables. This is a purely deterministic framework.

Authors' response: As stated in the manuscript, the equations used to model CV^2 (noise in expression) come from stochastic differential equations solved in the mean-field limit. We have included a supplementary Appendix (S1) in this revised version to present such a derivation. This has been done in several previous works, including some of ourselves. This is not a deterministic approach, but an analytical framework to study the noise. We suggest the reviewer to read Pedraza & van Oudenaarden, Science, 2005, 307:1965-1969; or Rodrigo, Phys. Rev. E, 2019, 100:032415.

2. The second term in these equations representing "intrinsic" noise assumes a constant Fano factor. Based on Friedman et al. (PRL 97: 168302), cited by the authors, and also Thattai and van Oudenaarden (PNAS 98: 8614) one would expect that the Fano factor is given by b+1 (discrete master equation) or b (continuous master equation). b is the average number of proteins produced per burst, which is proportional to the translation rate. If the translation rate of sfGFP is being regulated by MCP, the Fano factor (=b) should be dependent on the amount of MCP/eBFP2 and not a constant as the authors assume.

SCIENTIFIC PARK OF U. VAL.
9 CAT. AGUSTÍN ESCARDINO
46980 PATERNA, SPAIN

Authors' response: Analytically, it turns out that the intrinsic noise is (1+k2/d1)/(k1*k2/(d1*d2)), where k1 is the transcription rate, k2 the translation rate, d1 the mRNA degradation rate, and d2 the protein dilution rate (cell growth rate plus protein degradation rate). The term x = k1*k2/(d1*d2) is the mean protein expression, and b = k2/d1 is the burst size. When the repression occurs at the level of transcription, k1 is variable and k2 constant. Then, one can say that the Fano factor scales with the translation rate. However, when the repression occurs at the level of translation, k1 is constant and k2 variable. If the repression is strong, as it is the case here, b << 1, and the Fano factor can be approached by 1. This agrees with the experimental data. In addition, one must note that, when the expression changes as a result of k2, the plot of (1+b)/x vs. x in log scale does not reveal a linear trend if b >> 1, as k2 affects both b and x. We have elaborated this idea more in the manuscript to clarify the point.

3. Based on the above two points, the authors are effectively carrying out a deterministic curve fitting exercise when modeling the noise.

Authors' response: Analytical expressions for the noise can be derived (this is what we did). These expressions model the inherent stochasticity of the system, despite the kinetic parameters are adjusted by deterministic curve fitting. Of course, it is not possible to do it otherwise.

4. Another problem is the use of the terms "extrinsic" and "intrinsic". Barring a two-color experiment (two colors of the translationally repressed protein), it is not possible to decompose the noise into extrinsic and intrinsic components. Although one may decompose the total noise into two terms (intrinsic and extrinsic), decomposing further into three or more terms (each representing averages over subsets of independent variables, see Swain et al. PNAS 99: 12795) is not possible as far as I can tell. If the authors have done so, they should include a derivation. Third, the transfer function noise is also extrinsic since it involves the randomness of eBFP2 expression but not the inherent stochasticity of translation.

Authors' response: The decomposition of the total noise in different noise terms is a normal practice in the field. From a top-down perspective, the extrinsic noise is constant and the intrinsic noise scales inversely with the mean expression (see e.g. Hornung et al., Genome Res., 2012, 22:2409-2417; or Dar et al., PLoS One, 2016, 11:e0158298). This decomposition is possible when the expression level changes. The regulation introduces another component, which is conveniently separated from the two mentioned noise terms to perform the study. Noise propagation at the level of transcription was first studied in Pedraza & van Oudenaarden, Science, 2005, 307:1965-1969. Here, we study noise propagation at the level of translation for the first time with a synthetic gene circuit following the same approach.

The authors observed a lower level of noise in sfGFP but a clear mechanistic explanation has not been provided. The assumptions of a gene transcribed and translated at a constant rate at steady state match the experimental setup and the Gamma distribution describes the empirical histograms well. Then according to the results of Friedman et al. (PRL 97: 168302), CV^2 = 1/a (shown as Eq. 10 in Methods), where a is the Gamma distribution shape parameter that is proportional to the transcription rate (and not translation rate). This could imply that the lower noise in sfGFP is due to higher transcript numbers and not translational regulation.

SCIENTIFIC PARK OF U. VAL.
9 CAT. AGUSTÍN ESCARDINO
46980 PATERNA, SPAIN

Authors' response: In our manuscript we state that because the regulation occurs at the level of translation, transcription is constitutive, which contributes to have lower noise levels (lines 146-148, page 8, in previous version). We do not understand this comment because it is what we say. In any case, we have explained more this idea in the text.

The deterministic Hill equations used for determining the dependence of mean eBFP2 and sfGFP concentrations on IPTG and eBFP2 concentrations (Eq. 1) are unusual. The Hill equation for activation that results from mass kinetics takes the form (X/K)^n/(1 + (X/K)^n) and the Hill equation for repression takes the form 1/(1 + (X/K)^n). Equation 1 however combines both repression and activation:

alpha * (sigma + (IPTG/theta)^n)/(1 + (IPTG/theta)^n) = alpha * [sigma/(1 + (IPTG/theta)^n) + (IPTG/theta)^n/(1 + (IPTG/theta)^n)]

According to this equation IPTG both activates and represses eBFP2 simultaneously and sigma is a weight to determine the balance between activation and repression. This may have been done to achieve non-zero eBFP2 expression when there is no IPTG. A basal or leaky level of eBFP2 expression in the absence of IPTG might be better represented by adding a constant term to the Hill equation for activation.

Authors' response: With all due respect, we recommend the reviewer to do some maths, as the following equality holds

$$\frac{a + b\left(\frac{X}{K}\right)^n}{1 + \left(\frac{X}{K}\right)^n} = \frac{(b - a)\left(\frac{X}{K}\right)^n}{1 + \left(\frac{X}{K}\right)^n} + a\,.$$

The choice of a particular formulation is a matter of taste. We are not considering both repression and activation by IPTG, we are considering just activation. We suggest the reviewer to read e.g. Bintu et al., Curr. Opin. Genet. Dev., 2005, 15:116-124.

In summary, the paper treats propagation of noise in a deterministic manner and lacks any sort of stochastic treatment of translation noise under translational regulation.

Authors' response: We hope the reviewer, with this new version of our manuscript in hand, appreciates the analysis of the system in stochastic terms and finds it suitable for publication.

SCIENTIFIC PARK OF U. VAL.
9 CAT. AGUSTÍN ESCARDINO
46980 PATERNA, SPAIN
