# Supplementary material for: Gene regulation by a protein translation factor at the single-cell level
Source: PLoS Comput Biol. 2022 May 6;18(5):e1010087. doi: 10.1371/journal.pcbi.1010087 (PMC9116677; doi:10.1371/journal.pcbi.1010087)
Supplement: Attachment — Submitted filename: cover letter final revison.pdf [file pcbi.1010087.s011.pdf]

Valencia, 1 February 2022

Dear Editors of PLoS Computational Biology,

We are pleased to resubmit our manuscript entitled "Gene regulation by a protein translation factor at the single-cell level" (PCOMPBIOL-D-21-00828) for your consideration at PLoS Computational Biology.

Below, we provide a detailed point-by-point response to all reviewers' comments.

Thank you very much for your attention. I look forward to hearing from you.

Sincerely yours,

Guillermo Rodrigo, Ph.D.
Institute for Integrative Systems Biology (I2SysBio)
Consejo Superior de Investigaciones Científicas (CSIC)
Universitat de València (UV)
9 Cat. Agustín Escardino, 46980 Paterna, Spain
Phone: +34 963 544 786 (ext. 44786)
E-mail: guillermo.rodrigo@csic.es
Web: https://biosysdesign.csic.es

Reviewer #1

I would like to thank the Authors for responding to the earlier comments. Due to their edits, and due to their responses to both Reviewers' comments, the manuscript has improved substantially, and it has become more understandable. Now I would like to make some further comments, addressing which could make the manuscript acceptable for publication.

(1) Are there any natural systems what share the architecture of this synthetic system? It would be great to mention a couple of examples. That could increase the relevance of the research.

Authors' response: We have added the following text in the Discussion: "In prokaryotes, the regulation of translation initiation by controlling RBS accessibility is a widespread mechanism, but in eukaryotes the blockage of translation elongation has been observed in the case of Argonaute proteins [41]. Arguably, a blockage in the initial phase by MS2CP is key in our synthetic system." Ref. 41 corresponds to Iwakawa and Tomari (2013) Mol Cell 52: 591-601.

(2) The manuscript would become much clearer and more readable if the Results is divided into subsections, each with a subtitle. Possible subsections could have subtitles like 1: Constructing the experimental system; 2: Single cell expression measurements; 3: Deterministic and stochastic mathematical modeling; 4: Noise decomposition; 5: Modeling distributions; 6: The effect of translational inhibition.

Authors' response: We have divided the Results section with the following headers: i) Regulation of translation with an RNA-binding protein in single cells, ii) Noise analysis in transcription and translation regulation, iii) Examination of global effects on regulated gene expression, and iv) Integrative modeling of the deterministic and stochastic dynamics.

(3) Is there a native LacI gene in this E. coli strain? The lacI+ feature suggests this. How would native LacI expression affect the system? Likewise, there seems to be a tetR gene in the cells, as suggested by the tetR+ notation in the Methods. If TetR is expressed in the cells, it will bind Tetracycline (TC) and it will prevent translational inhibition by TC. That effect will contribute to the growth rate vs. TC profile in Figure 3C. Does TetR regulate any gene in the cells?

Authors' response: Yes, we are using a lacI+ strain. A native lacI expression would be insufficient to regulate a gene expressed under the PLlac promoter in a high copy number plasmid. The strain that we are using is also tetR+ (but TetR does not regulate any gene). This only means that the effective TC concentration will be slightly lower. We have clarified this in the Methods section.

(4) Error bars are not shown in figures, with the justification that the noise (CV^2, standard deviation, etc.) represents the error bars. However, this is not a standard way to omit error bars. Error bars should arise from biological replicates instead of single cell values deviating from the average. Therefore, all the mean and noise curves should have error bars representing the deviations of biological replicates from a mean. In other words, there should be a mean of the mean, a mean of the noise, a standard deviation of the mean and a standard deviation of the noise calculated from at least 3 biological replicates. Likewise, error bars around simulated values could arise from repeated stochastic simulations. See Murphy et al., PNAS 104 (31) 12726-12731 (2007) and Murphy et al., Nucleic Acids Res. 38(8): 2712–2726 (2010).

SCIENTIFIC PARK OF U. VAL.
9 CAT. AGUSTÍN ESCARDINO
46980 PATERNA, SPAIN

Authors' response: The single-cell measurements were repeated for three different populations (in the case of IPTG variation), finding consistency in the results. See the new Fig. S1.

(5) While the theoretical approach is interesting, it would be helpful to also try some stochastic simulations of the system. Such simulations might predict at least the intrinsic noise behaviors without TC.

Authors' response: As suggested, we have performed numerical simulations of the stochastic differential equations (see the new Fig. S2), finding good agreement with the analytical results.

(6) Adding TC will generate single-cell differences in cell division rate. The overall growth rate will be the average of all single cell division rates. In other words, there is a fitness average and a fitness noise. In the case of TC treatment, how large is the single-cell fitness noise? These concepts introduced in PLoS Comput. Biol. 8(4): e1002480 (2012) and then revisited in PLoS Comput Biol. 12(3): e1004825 (2016) and PNAS 115 (45) E10797-E10806 (2018) would be worth studying or at least discussing for TC. The fitness noise due to TC could be a source of extrinsic noise that correlates the extrinsic fluctuations of eBFP and sfGFP.

Authors' response: We thank the reviewer for this interesting comment. We have added the following text in the Discussion: "It is important to notice that the growth rate that we measured here corresponds to an average of the population (as we calculate it having monitored absorbance with time for a culture). Nevertheless, each cell grows differently, especially when the culture is under the effect of TC. In this regard, a mathematical model incorporating such heterogeneous growth might explain better the observed noise patterns [37]." Ref. 37 corresponds to Ray et al. (2016) PLoS Comput Biol 12: e1004825.

(7) In Equations (6), the constants for extrinsic noise are strong assumptions. Are these constants assumed the same for all IPTG and TC conditions? It may be an issue if TC affects dilution mean & noise.

Authors' response: We thank the reviewer for this comment. We have revised our model and now the extrinsic noise parameters scale with the inverse of the growth rate (i.e., more TC, more extrinsic noise). Thus, we have redone Figs. 4g,h,i. This is in tune with recent experiments characterizing genome-wide noisy expression levels. We have also cited the work Urchueguía et al. (2021) PLoS Biol 19: e3001491.

(8) There are some minor grammar issues that would be helpful to address. For example, in line 51: "pursuit" may be better; line 60: "progress has been made"; in line 119: "MS2CP being" would be better. Also, some clarifications would be useful, such as on line 150: "the total noise of both eBFP and sfGFP", on line 182: "inverse of the noise (equal to mean^2/variance)".

Authors' response: We have addressed all these points according to the referee's advice.

SCIENTIFIC PARK OF U. VAL.
9 CAT. AGUSTÍN ESCARDINO
46980 PATERNA, SPAIN

Reviewer #2

1. In response to the criticism that the manuscript appeared not to be performing stochastic modeling, the authors have clarified that equation 6 has been derived by modeling mRNA/protein concentration dynamics using the Langevin equation. Expressions for the variance of eBFP2 and sfGFP have been determined analytically by computing the power spectra and using the Wiener-Khintchine theorem.

I went back to the original submission and looked again and "Langevin" is not mentioned even once in the entire manuscript. The results state (lines 115-117 of original ms):

"We then constructed a mathematical model relying on a series of algebraic equations from basics on the biochemistry of gene expression and molecular noise propagation [7]"

This sentence can describe almost any model in computational biology. Even in the Methods section, which should describe the *methods* in sufficient detail so that the work may be reproduced, the sentence describing the model states: "The expressions for the variances in gene expression (or fluorescence signal) can be derived by following some basic calculations [3,7]."

If the authors do not specify what "basic calculations" were performed, the readers, including the reviewer, are free to use their imaginations. As it happens, the same expressions for variance can be derived by error propagation, with very similar assumptions/approximations (sources of noise are independent, linear approximation).

Authors' response: We have specified in the Methods section that "We followed a Langevin formalism, which consists in introducing in the right-hand side of the differential equation a series of stochastic processes, and the mean-field approximation for the analytical treatment, which consists in assuming that the different fluctuation amplitudes depend on the deterministic solution." And we agree with the referee that our initial version of the manuscript was scarce in mathematical details, which are now included in the Appendixes.

The authors have included a derivation of equation 6 in Appendix S1 of the revised manuscript. The authors still fail to mention/describe the modeling method or its underlying assumptions, apart from the very generic descriptions quoted above, in the results or methods. The reader should not have to refer to other papers or read the appendices to know at a high level the method used to arrive at equation 6, which is central to the analysis presented in the paper. This is also important for critically assessing the manuscript. For example, one crucial assumption is that the noise sources are independent. This is not strictly true since eBFP2 and sfGFP share extrinsic noise sources and so the regulation noise from eBFP2 is not independent of extrinsic noise. In this, the manuscript appears to diverge from the treatment in Pedraza and van Oudenaarden (Science, 307:1965), who modeled transmitted noise as strongly correlated with global (equivalent to extrinsic in this ms's terminology). These assumptions were not mentioned at all in the original submission and are only mentioned in the appendix in the revised version.

The authors should include a short description of the Langevin formalism and its assumptions in the results and methods sections. They have already described how they chose the variances for the different noise terms (lines 418-424). A description of the analytical methodology and its assumptions is missing. It would also be useful to discuss the limitations imposed by these assumptions in the Discussion section.

Authors' response: We have included this text in the Results section: "To derive these mathematical expressions for the mean and the noise, we constructed a system of

SCIENTIFIC PARK OF U. VAL.
9 CAT. AGUSTÍN ESCARDINO
46980 PATERNA, SPAIN

stochastic differential equations for mRNA and protein expression following the Langevin formalism. The rates of concentration changes were subject to stochastic fluctuations of intrinsic and extrinsic nature. This system was analytically solved in steady state with the mean-field approximation for the fluctuations."

In addition, we have included this text in the Discussion: "Another limitation of our work is the assumption that the noise sources are independent, which allowed us to derive a compact mathematical expression for the noise of sfGFP. This is not strictly true since eBFP2-MS2CP and sfGFP share extrinsic noise sources [7]. Yet, we expect reliability in the conclusions derived from this study".

2. The lack of sufficient description of the modeling also explains the confusion regarding the terms "intrinsic" and "extrinsic". The impression one gets from the manuscript (lines 149-151 of revised ms): "For that, we first decomposed the total noise into three fundamental components: extrinsic noise, intrinsic noise, and regulation noise."

is that it is *always* possible to decompose the noise into three components, which is not correct. Without any further assumptions, noise can be decomposed into two components (Swain et al. PNAS 99: 12795) but not three. To achieve the decomposition carried out in the ms, it is necessary to make the additional assumption that "extrinsic", "intrinsic", and regulatory random variables are independent. A better wording might be something like:

"We modeled stochastic mRNA/protein expression using Langevin equation. The rate of change of mRNA concentration was subject to fluctuations from three sources: .... Assuming that the sources are independent, the noise can be decomposed into three contributions"

Authors' response: We have introduced the following text in the Results section: "In previous work, the regulation noise has been considered as a part of the extrinsic noise. Here, we separate this component to study more in detail the stochastic gene expression when it is regulated. Assuming independence between the different stochastic sources, we were able to end with compact mathematical expressions for the noise in which the different components were identified, although at the cost of introducing some inaccuracies since the extrinsic noise may correlate responses."

The authors have cited Pedraza and van Oudenaarden (Science, 307:1965), who defined the noise sources more carefully as global, intrinsic, and plasmid. The authors should consider following that example since "extrinsic" means all sources that are not inherent to the transcription/translation process (Elowitz et al., Science 297:1183, Swain et al. PNAS 99: 12795) and includes, among other things, transmitted noise.

Authors' response: We agree with the reviewer that according to Elowitz and colleagues extrinsic noise includes all that is not intrinsic noise. For clarification purposes, we have stated that "In previous work, regulation noise has been considered as a part of the extrinsic noise."

3. The authors have satisfactorily addressed all the other points raised in the review.

Authors' response: We thank the reviewer for this appreciation.

4. A general comment: part of the difficulty of understanding the manuscript has been that it has been written very tersely and is stingy with explanations, yet it shies away from

http://i2sysbio.uv.es

SCIENTIFIC PARK OF U. VAL.
9 CAT. AGUSTÍN ESCARDINO
46980 PATERNA, SPAIN

technical/mathematical descriptions in the main text. Anything the authors can do to expand their explanations would help.

Authors' response: In this new version of the manuscript, we have included several sentences that aim at clarifying different points of our work.

For example, it is indeed true that the authors noted that the lower noise in sfGFP must be due to a high transcription rate (lines 146-148, original submission), but it is stated in such an off-hand manner: "This suggested that with a translational control the noise of the regulator is buffered, as the regulated gene is transcribed at high levels."

and without reference to the theoretical results that it was easily missed.
It would be better to expand this explanation a bit and point out that 1) the noise is inversely related to the shape parameter of the Gamma distribution and 2) that the shape parameter is proportional to the transcription rate (ideally using the mathematical expressions).

Authors' response: We expanded the Results section with the following text in our previous revision: "This suggested that with a translational control the noise of the regulator is buffered, i.e., the fluctuations of MS2CP expression are manifested on sfGFP expression in lower extent than the fluctuations of LacI expression on MS2CP expression. This is because in a scenario of translational control the regulated gene is constantly transcribed at high levels, where fluctuations in the number of mRNAs per cell are small in comparison with the mean quantity that is produced. Thus, the transcriptional noise is not significant. In addition, the regulation enters at the level of translation, which prevents the typical amplification process of the noise of the regulator that occurs with a transcriptional control".

Now we have also added the following text in the Results section: "As a result of a transcriptional control, the Gamma scale parameter for eBFP2-MS2CP depends on the translation rate; but in the case of sfGFP, as this element is controlled at the level of translation, the Gamma scale parameter is nearly independent of that rate."
